# Lupus Retinopathy Mimicking Ocular Tuberculosis: A Diagnostic Dilemma

**DOI:** 10.7759/cureus.111371

**Published:** 2026-06-23

**Authors:** Supriya Rawat, Suhani Malhotra, Priksha Lakhlan, Shrutanjoy Das, Vaibhav Bhatt, Gaurav Luthra, Subodh Gururani, Vijay Bakaya, Saurabh Luthra

**Affiliations:** 1 Ophthalmology, Drishti Eye Institute, Dehradun, IND; 2 Rheumatology, Shri Guru Ram Rai Institute of Medical and Health Sciences (SGRRIMHS) and Shri Mahant Indiresh Hospital (SMIH), Dehradun, IND; 3 Dermatology, Dr. Bakaya's Skin and Aesthetic Clinic, Dehradun, IND

**Keywords:** branch retinal artery occlusion, chorioretinitis, lupus retinopathy, neuropsychiatric sle, ocular tuberculosis, retinal vasculitis

## Abstract

Systemic lupus erythematosus (SLE) or Lupus is often called the “great mimicker” due to its ability to mimic other systemic disorders. SLE is a multisystem autoimmune disorder with varied manifestations, including ocular involvement. Retinal vascular occlusions are an uncommon but potentially vision-threatening presentation of lupus retinopathy. When coexisting systemic features mimic infectious etiologies such as tuberculosis (TB), diagnosis becomes challenging, delaying appropriate immunosuppressive therapy.

We report a diagnostically complex case of a 22-year-old female who presented with sudden, painless loss of vision in the right eye. Initially, she was diagnosed elsewhere as macular branch retinal artery occlusion (BRAO) and chorioretinitis. Systemic symptoms-weight loss, low-grade fever, and neuropathy-along with a positive sputum smear and chest radiograph findings, led to a provisional diagnosis of pulmonary tuberculosis with presumed ocular TB. She was started on anti-tubercular therapy (ATT) and oral corticosteroids. Upon further evaluation at our center, additional ocular findings included disc pallor, active vasculitis, and bilateral chorioretinitis. Imaging studies (Fundus Fluorescein Angiography (FFA), Indocyanine Green Angiography (ICGA), Optical Coherence Tomography (OCT)) confirmed retinal vasculitis. A dermatologic evaluation of her facial rash was inconclusive for SLE. However, subsequent worsening of systemic symptoms and neuropsychiatric events prompted re-investigation. Specific MRI findings and immunological tests that revealed strong positivity for anti-Smith, anti-ribonucleoprotein (anti-RNP), anti-Ro-52, and anti-double-stranded deoxyribonucleic acid (anti-dsDNA) antibodies with hypocomplementemia confirmed the diagnosis of neuropsychiatric SLE (NPSLE). ATT was discontinued, and the patient was treated with intravenous steroids and cyclophosphamide and later transitioned to mycophenolate mofetil. Over a year, the patient showed significant systemic and ocular improvement, with best-corrected visual acuity in the affected eye improving from perception of light to 6/60.

This case highlights the diagnostic dilemma in distinguishing retinal vasculitis of autoimmune etiology from that due to infectious causes such as tuberculosis, especially in endemic regions. Ocular involvement can be an early sign of systemic SLE, underscoring the need for high clinical suspicion, systemic correlation, and immunological workup in young patients with retinal vascular occlusions. Early recognition of lupus retinopathy and timely immunosuppression are key to preserving vision and preventing systemic morbidity.

## Introduction

Systemic lupus erythematosus (SLE) is a chronic autoimmune disease with protean manifestations that can affect multiple organ systems, including the eye. About 20-33% of SLE patients report ocular manifestations, including abnormalities of the adnexa, cornea, iris, retina, choroid, and optic nerve [1]. Lupus retinopathy is the second most common ocular manifestation, the most common being dry eye. Lupus retinopathy has more significance due to its association with systemic disease activity and prognosis. Moreover, retinopathy might be an early manifestation of the disease [2]. Retinopathy in SLE typically presents as three distinct clinical phenotypes [3]. The most prevalent form is lupus-associated retinal microangiopathy, which arises due to immune complex deposition within the retinal vasculature, leading to focal ischemia, seen clinically as cotton-wool spots, microaneurysms, and intraretinal hemorrhages [1,4]. Another manifestation is retinal vasculitis, which is characterized by perivascular sheathing, vascular wall thickening, and infiltration of inflammatory cells. It indicates active ocular and systemic inflammation and often coexists with central nervous system involvement [5,6]. The most fulminant phenotype is thrombo-occlusive retinopathy, which may present as central or branch retinal artery or vein occlusions. However, the fundoscopic features of lupus retinopathy are not pathognomonic and may mimic other inflammatory or infectious retinal conditions, including Behcet’s disease, idiopathic vasculitis, syphilis, cytomegalovirus retinitis, and ocular tuberculosis (TB) [7].

Ocular involvement in tuberculosis occurs in approximately 1.4% to 10.5% of cases in regions with a high TB burden, whereas it is encountered far less frequently in countries with low TB incidence [8]. Clinical manifestations include retinal involvement, typically choroidal inflammation (chorioretinitis) and retinal vasculitis (perivascular infiltrates, vascular occlusions, and subvascular lesions). While venular and arterial involvement has been observed in Ocular TB, a patient presenting with branch retinal artery occlusion (BRAO) is a rare primary manifestation of SLE retinopathy. This can lead to a diagnostic challenge, particularly in regions endemic for TB, where overlapping signs such as vascular sheathing and choroiditis may make clinicians lean towards an infectious etiology. We present such a case of atypical lupus retinopathy initially misdiagnosed as presumed ocular TB in a setting of a highly TB-endemic country.

## Case presentation

**Figure 1:**
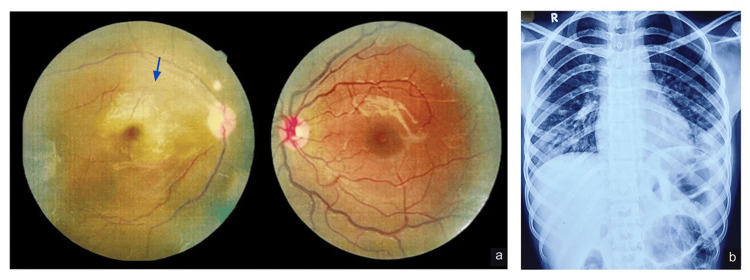
Imaging results performed at the referring centre (a) Fundus photograph done one month before presentation to our hospital suggestive of OD acute presentation of Branch Retinal Artery Occlusion (BRAO) involving macula with Cherry Red Spot (blue arrow), OS vascular tortuosity. (b) X-ray Chest showing small patches of fluffy radio opacities in bilateral lung fields sparing upper lung fields suggestive of small air space consolidation/pneumonitis, likely due to infective etiology. OD - Right eye, OS - Left eye.

A 22-year-old asian female from a tuberculosis-endemic region was referred to us with the complaint of diminution of vision in the right eye for the past two months, which was painless and sudden in onset (Month 2). She was diagnosed elsewhere as right eye macular branch retinal artery occlusion (BRAO) with a cherry red spot and chorioretinitis (Month 0) (Figure 1).

In view of the BRAO, a systemic evaluation was undertaken to identify potential underlying etiologies (Month 0). Serum homocysteine levels were 5.80 µmol/L (reference range 5.3-16.5 µmol/L), ruling out hyperhomocysteinemia as a contributing factor. Carotid Doppler ultrasonography showed no evidence of atherosclerotic plaques or flow-limiting stenosis, thereby excluding significant carotid artery disease as an embolic source.

The patient also underwent detailed investigations at the referring center in view of persistent systemic symptoms, *viz.,* evening rise in temperature, loss of appetite, significant weight loss, hair fall, lower-limb pain, and generalized weakness over the preceding year. Laboratory workup revealed subclinical hypothyroidism, with an elevated thyroid-stimulating hormone (TSH) level of 6.65 μIU/mL (reference range 0.20-5.00 μIU/mL). Hematological analysis demonstrated anemia (hemoglobin 7.2 g/dL), elevated total leukocyte count (12,600/cu mm), neutrophilia (77%), and lymphopenia (17%). Liver function tests, renal function tests, echocardiography, and ultrasound of the abdomen and pelvis were all normal. Enzyme-linked immunosorbent assay (ELISA) for HIV and Venereal Disease Research Laboratory (VDRL) tested negative. Inflammatory markers were significantly elevated, with an erythrocyte sedimentation rate (ESR) of 129 mm in the first hour (reference range 0-20 mm/1st hr) and a C-reactive protein (CRP) level of 82.3 mg/dL (reference range 0-0.50 mg/dL), suggesting an ongoing systemic inflammatory process. Rheumatoid (RA) factor was normal (4.12 IU/mL (reference range 0.00-20.0 IU/mL), serum anti-nuclear antibody (ANA) was negative at 0.68 U (positive > 1.0 U) (Table 1).

**Table 1:** Laboratory test results at the referring centre TSH - Thyroid-stimulating hormone, ELISA - Enzyme-linked immunosorbent assay, VDRL - Venereal disease research laboratory, ESR - Erythrocyte sedimentation rate, CRP - C-reactive protein, RA - Rheumatoid arthritis, ANA - Antinuclear antibody.

Investigation	Value	Reference range
Serum Homocysteine	5.8	5.3-16.5 µmol/L
TSH	6.65	0.20-5.00 μIU/mL
Hemoglobin	7.2	11.5-15.5 g/dl
Total Leucocyte Count	12,600	4000-11,000 /cu mm
Differential Leukocyte Count		
1) Neutrophils	77	50-70%
2) Lymphocytes	17	35-40%
3) Eosinophils	1	1-6%
4) Monocytes	5	2-10%
HIV (ELISA)	Negative	
VDRL	Negative	
ESR	129	0-20 mm/1st hr
CRP	82.3	0-0.50 mg/dL
RA Factor	4.12	0.00-20.0 IU/mL
ANA	0.68	Positive > 1.0 U

Given the systemic symptoms, abnormal chest radiograph findings (Figure 1b), and sputum acid-fast bacillus (AFB) 1+ positive report on Ziehl-Neelsen staining, suggestive of pulmonary Koch’s disease and suspected Ocular tuberculosis (TB), the patient was empirically initiated on anti-tubercular therapy (ATT) along with systemic corticosteroids. To further assess the patient’s neurological complaints, a nerve conduction velocity (NCV) study was conducted. The findings were consistent with a mild degree of sensory-motor axonal neuropathy involving the left peroneal nerve, as well as a mild motor axonal neuropathy affecting the left tibial and right peroneal nerves.

**Figure 2:**
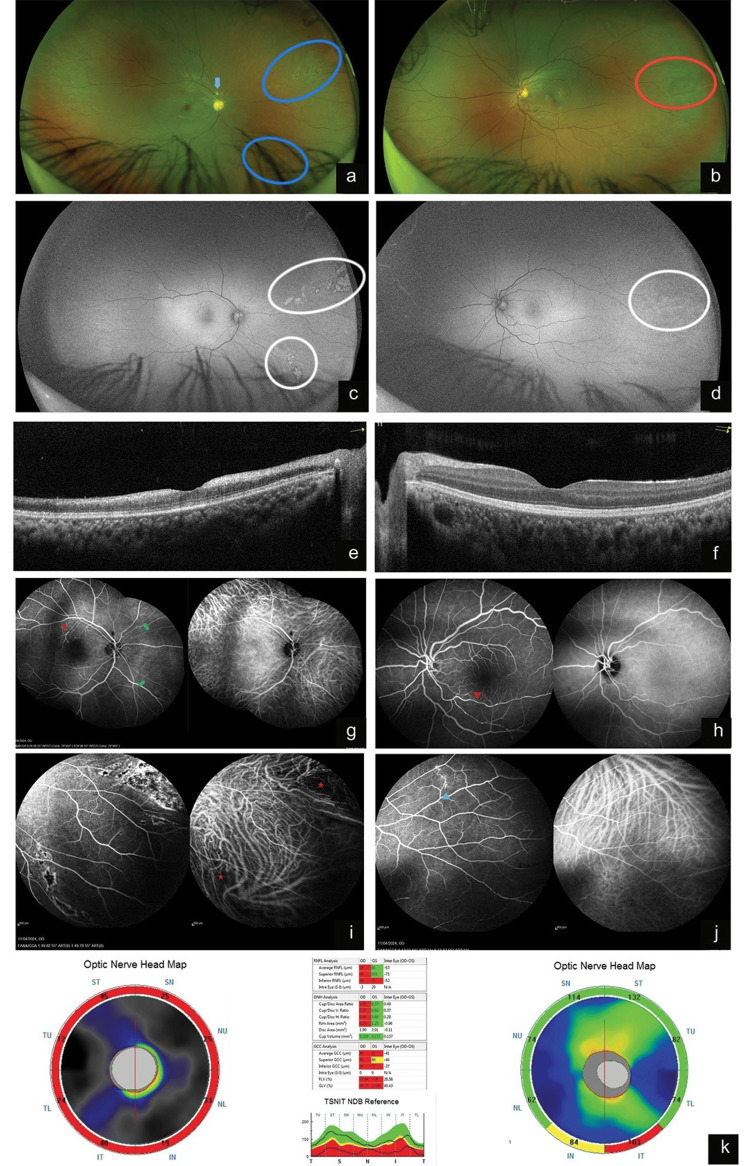
Multimodal imaging of both eyes at presentation to our centre (a and b) UWF imaging showing OD Disc pallor, isolated exudate superior to disc (blue arrow), superonasal and inferonasal chorioretinitis patches (blue circles), OS tortuous vessels and chorioretinitis lesion in temporal periphery (red circle). (c and d) UWF Fundus Autofluorescence (FAF) imaging OU showing peripheral hyperautofluorescent lesions with hypoautofluorescent margins (white circles) corresponding to chorioretinitis patches on color imaging. (e and f) Spectral Domain Optical Coherence Tomography (SD-OCT) of macula showing OD Inner Retinal Layer (IRL) hyperreflectivity, OS showing normal anatomy. (g - j) FFA and ICGA imaging - (g) OD showing occlusive vasculitis in the distal superotemporal macular arterial branch (red arrow on FFA) with proximal arterial narrowing, pruning of vessels nasally (green arrows on FFA). (h) OS showing cattle trucking of inferotemporal branch of retinal artery (red arrowhead on FFA). (i) FFA OD showing peripheral chorioretinitis patches with hypofluorescent centre and hyperfluorescent margins, hypocyanescence on ICGA (red asterisk). (j) OS showing leakage from distal tortous vessels in temporal periphery on FFA (blue arrowhead). (k) SD-OCT Retinal nerve fibre layer (RNFL) OU showing OD gross RNFL loss and OS inferior RNFL thinning. OD - Right eye, OS - Left eye, UWF - Ultra-widefield, OU - Both eyes, FFA - Fundus Fluorescein Angiography, ICGA - Indocyanine Green Angiography

At presentation to our center (Month 2), the patient appeared to be cachexic (body weight 30 kg). She had faint rashes over her face. Ocular examination revealed right eye exotropia. Best-corrected visual acuity (BCVA) was perception of light (PL+) with projection of rays (PR) defective in the right eye and 6/6, N6 in the left eye. Intraocular pressure (IOP) was within normal limits in both eyes. Relative afferent pupillary defect was present in the right eye. Anterior segment evaluation showed no anterior chamber reaction. However, the anterior vitreous face showed vitreous cells 2+ in the right eye and 1+ in the left eye on slit-lamp biomicroscopy. Fundus examination (Daytona Optos®, Dunfermline, Scotland, UK) revealed right eye optic disc pallor, resolving exudate superior to the disc, resolving superotemporal BRAO involving the macula, and healing chorioretinitis lesions in the superonasal and inferonasal midperiphery (Figure 2a). Left eye revealed venous dilatation and tortuosity, focal areas of vasculitis along the inferotemporal arcade, and healing chorioretinitis lesions in the temporal periphery (Figure 2b). Spectral domain optical coherence tomography (SD-OCT) (RTVue XR Avanti, Optovue Inc., Fremont, CA, USA) showed right eye inner retinal hyperreflectivity and thinning at the macula, while the left eye was normal (Figures 2e, 2f). Fundus fluorescein angiography (FFA) (Spectralis Heidelberg Retina Angiograph (HRA), Heidelberg, Germany) of the right eye revealed narrowing of the proximal segment of the superotemporal branch retinal artery supplying the macula and fluorescein staining of the distal segment of the same vessel (Figure 2g). FFA of the left eye showed cattle trucking along the inferotemporal arcade suggestive of occlusive vasculitis (Figure 2h). FFA of the right eye also showed staining of chorioretinitis lesions in the superonasal and inferonasal periphery, while Indocyanine Green Angiography (ICGA) (Spectralis Heidelberg Retina Angiograph (HRA), Heidelberg, Germany) revealed hypocyanescence of these chorioretinitis patches (Figure 2i). FFA of the left eye showed leakage from a tortuous vessel in the superotemporal mid periphery suggestive of active vasculitis (Figure 2j). SD-OCT Retinal nerve fiber layer (RNFL) of the right eye revealed significant RNFL loss in all sectors due to optic atrophy, and the left eye showed inferior RNFL thinning (Figure 2k).

Based on the endemic setting, systemic symptoms, and prior investigations, along with the absence of an alternative etiology, a diagnosis of Presumed Ocular tuberculosis (TB) was made in accordance with Collaborative Ocular Tuberculosis Study (COTS) criteria. Intraocular fluid PCR or culture for Mycobacterium tuberculosis was not performed due to resource constraints. Following the initial evaluation, the patient was started on topical steroid and topical antibiotic eye drops and sent for systemic evaluation and laboratory investigations. Complete blood count (CBC) revealed anemia (Hb 8.1 g/dl) (reference range 11.5-15.5 g/dl) but normal total leucocyte count. General blood picture (GBP) revealed a dimorphic anemia (both macrocytic and microcytic hypochromic red blood cells) along with mild thrombocytopenia. Additionally, inflammatory markers like ESR were raised, i.e., 27 mm/hr (reference range 9-20 mm in 1st hour), but CRP was normal, i.e., 1.8 mg/L (reference range 0-5mg/L). Serum ANA using immunofluorescence on human epithelial type 2 (HEp-2) (cutoff titer 1:40) was negative (Table 2).

**Table 2:** Laboratory test results performed at our facility ESR - Erythrocyte sedimentation rate, CRP - C-reactive protein, ANA - Antinuclear antibody, AST - Aspartate aminotransferase, ALT - Alanine aminotransferase.

Investigation	Value	Reference range
Hemoglobin	8.1	11.5-15.5 g/dl
Total Leucocyte Count	7500	4000-11,000 /cu mm
Absolute Leucocyte Count		
1) Neutrophils	2570	2000-7000 /µL
2) Lymphocytes	960	1000-3000 /µL
3) Eosinophils	80	20-500 /µL
4) Monocytes	20	20-1000 /µL
ESR	27	9-20 mm in 1st hour
CRP	1.8	0-5 mg/L
Serum ANA	Negative	cutoff titre 1:40
Serum AST	42	0-32 U/L
Serum ALT	39	0-33 U/L

**Figure 3:**
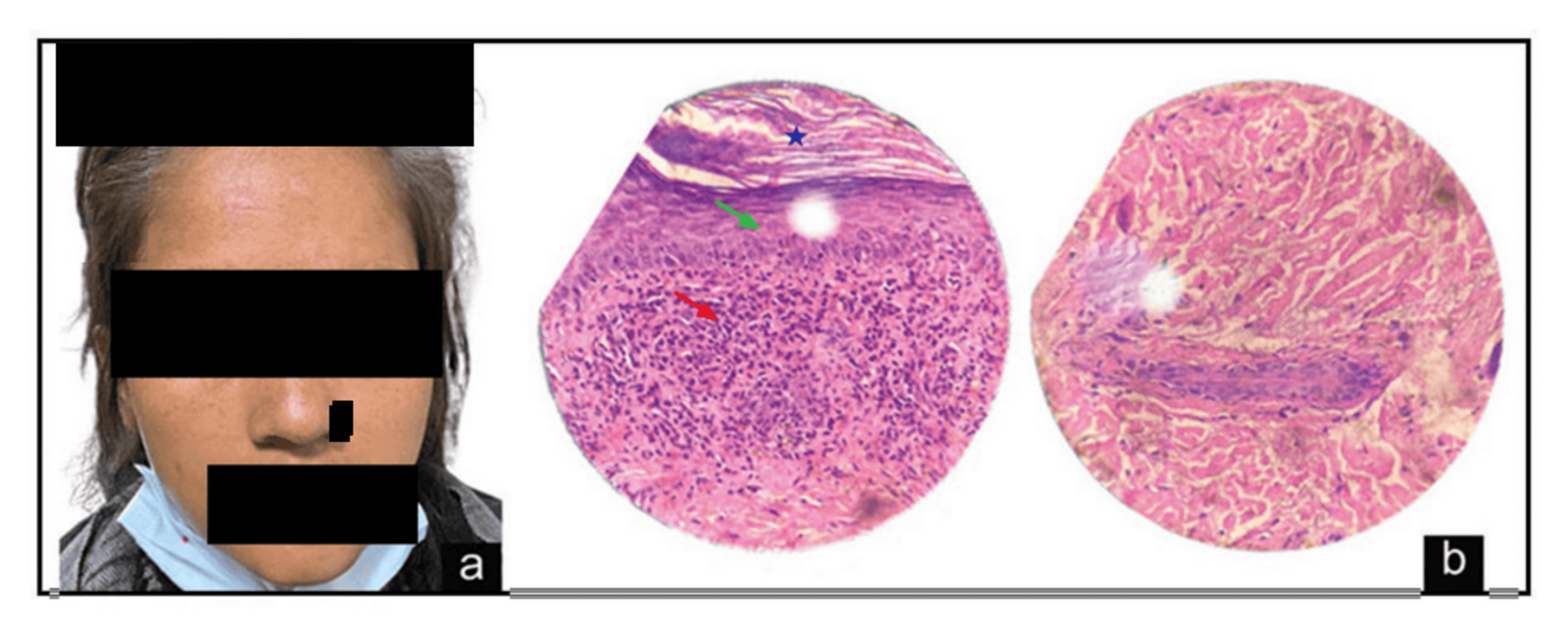
Systemic evaluation at first presentation (a) Faint rashes over the forehead and nose can be seen, (b) photos of histopathology slides from skin biopsy showing mild spongiosis of the epidermis with focal parakeratosis (blue asterisk) with few necrotic keratinocytes, minimal vacuolar degeneration of the basal layer (green arrow), and extravasation of red blood cells in the epidermis (red arrow). Papillary dermis showing edema, dilated capillaries, macrophages, erythrocytes extravasation with mild perivascular lymphocytic infiltration.

In view of the rash over the face, the patient was referred to a dermatologist. She was found to have faint pinkish flat rashes over the forehead and nose (Figure 3a). A skin biopsy from the rash was taken and sent for histopathology and direct immunofluorescence. Histopathology was suggestive of mild spongiosis of the epidermis, focal parakeratosis, a few necrotic keratinocytes, and minimal vacuolar degeneration of the basal layer with extravasation of red blood cells in the epidermis. Papillary dermis showed edema, dilated capillaries, melanophages, erythrocyte extravasation with mild perivascular lymphocytic infiltration. No fibrinoid necrosis of the vessel wall was seen. Immunofluorescent study showed focal deposits of immunoglobulin (Ig)M and Complement Component 3 (C3) at the basement membrane zone, with no deposition of IgG, IgA, or vascular immune complexes. These findings were interpreted as not supportive of SLE histopathologically but were more suggestive of Pityriasis Lichenoides Chronica/Acuta (Figure 3b).

**Figure 4:**
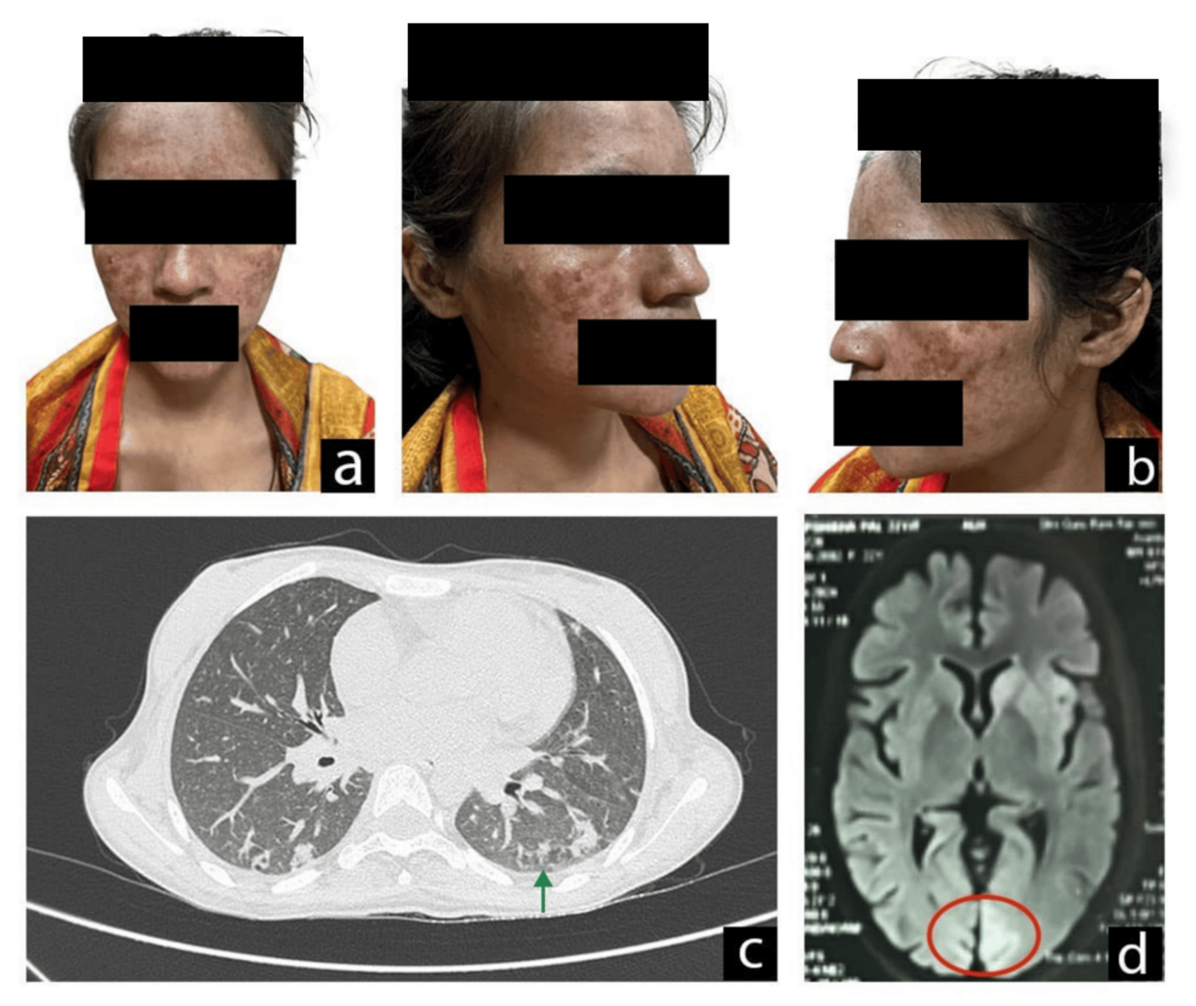
During the episode of systemic exacerbation (a and b) Facial profile of the patient, (c) high-resolution computed tomography (HRCT) of the thorax showing a nonspecific interstitial pneumonitis (NSIP) pattern (green arrow). (d) MRI brain image showing near symmetrical areas of faint signal alteration in bilateral occipital regions suggestive of Posterior Reversible Encephalopathy Syndrome (PRES) (red circle).

Two months later (Month 4), the patient was admitted to a multispecialty hospital with worsening systemic symptoms and facial rash (Figures 4a, 4b). Based on the symptoms of photosensitivity, facial swelling, erythematous rash over bilateral cheeks, evening rise of temperature persisting for over seven months, loose stools, arthralgia, generalized weakness, and retinal vasculitis, she was clinically diagnosed as a case of SLE. A pulmonology consultation was obtained in view of an initial diagnosis of pulmonary TB. High-resolution computed tomography (HRCT) of the thorax revealed a nonspecific interstitial pneumonitis (NSIP) pattern (Figure 4c). Bronchoscopy was unremarkable. Bronchoalveolar lavage (BAL) fluid analysis was negative for malignant cells and fungal elements, and bacterial as well as fungal cultures showed no growth. Gram staining showed Gram-negative bacilli, but no evidence of AFB was found. Hence, ATT was discontinued.

A rheumatology consult was also taken, and serum ANA was re-evaluated, which was highly positive (1:3200 titers on immunofluorescence on HEp-2). ANA profile revealed: anti-Smith antibody, anti-ribonucleoprotein (RNP) antibody, anti-Sjögren's-syndrome-related antigen A (SS-A)/ RO-52 antibody, and anti-Ribosomal P (Rib-P) antibody all strongly positive; anti-double-stranded deoxyribonucleic acid (dsDNA) antibody positive; and borderline positive results for anti-Histone and anti-mitochondrial M2 (AMA-M2) antibodies. Also, hypocomplementemia was noted with serum C3 at 31.30 mg/dL, serum Complement Component 4 (C4) at 3.10 mg/dL (reference range: serum C3 84-168 mg/dL, serum C4 15-45 mg/dL) (Table 3). Based on these findings, she was initiated on pulse intravenous methylprednisolone 1 g/day for three doses followed by oral prednisolone 30 mg once daily, sulfasalazine 1 g twice daily, and supportive medications.

**Table 3:** Laboratory test results during systemic worsening ANA - Antinuclear antibody, Anti-Sm Ab - Anti-Smith antibody, Anti-Rib-P Ab - Anti-ribosomal P protein antibody, Anti-RNP Ab - Anti-ribonucleoprotein antibody, Anti-SS-A/RO-52 Ab - Anti-Sjögren's-Syndrome-Related Antigen A / 52 kDa Protein Antibodies, Anti-dsDNA Ab - Anti-double stranded DNA antibody, Ab - Antibody, AMA-M2 Ab - Anti-Mitochondrial Antibody, M2 subtype.

Investigation	Value	Reference range
Serum ANA	(1:3200)	≥1:80 (on IF on Hep-2)
ANA Profile		
1) Anti-Sm Ab	strong positive	-
2) Anti-Rib-P Ab	strong positive	-
3) Anti-RNP Ab	strong positive	-
4) Anti-SS-A/RO-52 Ab	strong positive	-
5) Anti-dsDNA Ab	positive	-
6) Anti-histone Ab	borderline positive	-
7) AMA-M2 Ab	borderline positive	-
Serum C3	31.3	84-168 mg/dL
Serum C4	3.1	15-45 mg/dL

During the course of hospitalization, the patient developed an episode of generalized tonic-clonic seizures, raising suspicion of central nervous system involvement. Magnetic Resonance Imaging (MRI) of the brain with contrast was ordered, which revealed near-symmetrical faint signal alterations on T2-weighted Fluid-Attenuated Inversion Recovery (T2/FLAIR) hyperintensities in bilateral occipital regions, with a diagnosis of Posterior Reversible Encephalopathy Syndrome (PRES) (Figure 4d). Cerebrospinal fluid (CSF) analysis showed no significant findings. Based on the neuro-ophthalmic findings, seizure episode, MRI Brain findings and systemic features, a diagnosis of neuropsychiatric SLE (NPSLE) was made. She was initiated on six doses of intravenous cyclophosphamide 500 mg at monthly intervals. Laboratory tests at this point showed stable parameters (Hb 12.4 g/dL, platelet count 150 ×10⁹/L, serum ALP 52 U/L, serum AST 25 U/L, serum ALT 33 U/L, serum albumin 3.9 g/dL, serum bilirubin 0.4 mg/dL, GBP normocytic normochromic). One month later, the patient was hospitalized again for anxiety, chest heaviness, and low-grade fever. A psychiatry consultation was done, and treatment was initiated for SLE-associated psychiatric symptoms.

Subsequently, she was maintained on oral mycophenolate mofetil (MMF) 500 mg twice daily, sulfasalazine 1 g twice daily, levetiracetam, and pregabalin, while oral steroids were gradually tapered and discontinued over the course of one year. At the four-month follow-up, there was an improvement in general condition. BCVA improved to 3/60 in the right eye, and there was resolution of ocular findings in both eyes.

**Figure 5:**
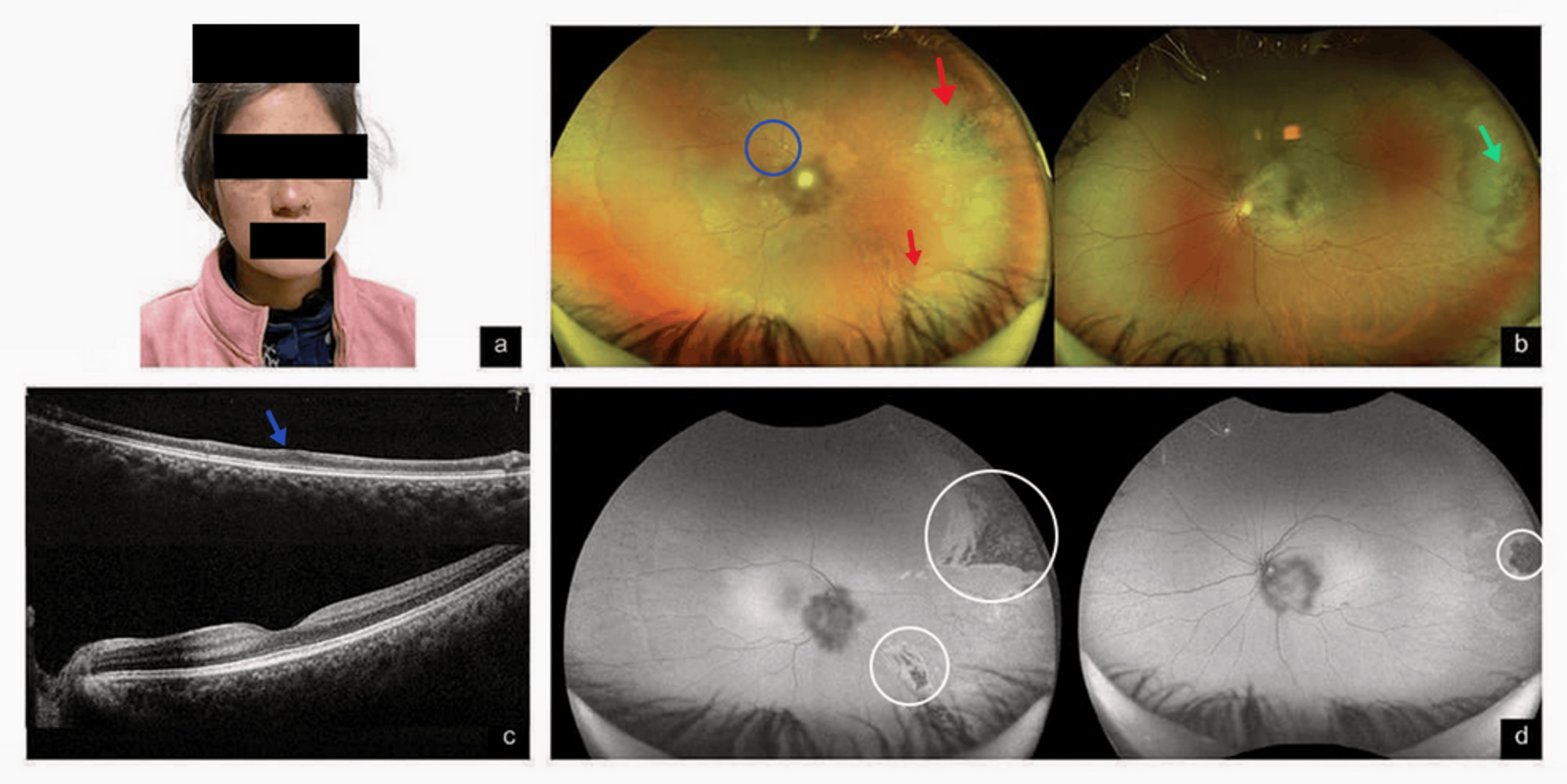
Clinical images at one year follow-up (a) Patient profile (one year post initiation of six doses of Injection cyclophosphamide & subsequent oral immunosuppressive treatment) showing resolution of malar rash over cheeks and forehead (b) UWF imaging depicting OU central media opacity due to PSC cataract; OD Optic atrophy, sclerosed arteries (blue circle) and chorioretinal atrophy patches (red arrows); OS resolved vaso-occlusive vasculitis and chorioretinal atrophy patches in periphery (green arrow). (c) Spectral Domain Optical Coherence Tomography (SD-OCT) of macula showing OD gross retinal thinning (blue arrow), OS showing normal anatomy. (d) UWF Fundus Autofluorescence Imaging OU showing peripheral healed chorioretinitis evident as hypoautofluorescent areas (white circles). UWF - Ultra-widefield, OU - Both eyes, PSC - posterior subcapsular, OD - Right eye, OS - Left eye.

One year post-initiation of systemic treatment for SLE, remarkable improvement (Figure 5) was observed in the facial rash (Figure 5a). BCVA right eye improved to 6/60, left eye was stable at 6/6, N6; and slit lamp examination showed posterior subcapsular cataract in both eyes. Fundus examination revealed disc pallor in the right eye and healed chorioretinitis lesions in both eyes (Figure 5b). SD-OCT of the macula showed right eye gross retinal thinning and left eye normal anatomy (Figure 5c).

## Discussion

SLE is a prototypical multisystem autoimmune disorder, exhibiting a striking predilection for females with a female-to-male ratio nearing 9:1 [9]. The disease may present with a wide spectrum of clinical manifestations ranging from mild cutaneous involvement to severe multiorgan dysfunction. One such organ is the eye, with SLE retinopathy as one of the most common presentations of active disease. Retinal damage in SLE can manifest in three primary forms: lupus microangiopathy, presenting with features such as cotton-wool spots, hard exudates, and retinal hemorrhages; retinal vasculitis, involving inflammatory changes in the larger retinal vessels and severe vaso-occlusive retinopathy, which may result in widespread retinal ischemia.

The pathophysiology underlying such presentations is either directly via immune complex-mediated vasculopathy or indirectly via secondary hypertension due to renal involvement [1]. Choroidopathy, though an uncommon presentation, indicates an underlying choroidal vasculitis [10]. Most of these cases present with bilateral neurosensory detachment, retinal pigment epithelium detachment, or retinal pigment epitheliopathy [1,3]. The most severe form of lupus retinopathy manifests as widespread retinal ischemia, ranging from occlusion of major vessels, such as the central retinal artery, central retinal vein, and cilioretinal artery, to diffuse microvascular embolization, as is characteristic of Purtscher-like retinopathy [7]. Vaso-occlusive retinopathy, in the form of branch retinal artery occlusion, is extremely rare in SLE and often leads to poor visual outcomes [1]. To the best of our knowledge, only one case of BRAO as the primary manifestation of SLE retinopathy has been reported in the past [11].

Central nervous system (CNS) involvement in SLE has been estimated to be 10.3-36.9% [12]. Parallels have been drawn between retinal vasculitis and NPSLE, considering the eye as a window to detect systemic involvement [3]. Lupus retinopathy is widely accepted as a clinical indicator of severe SLE, signifying widespread vascular injury and elevated disease activity [7]. NPSLE includes involvement of the central, peripheral, autonomic nervous systems, as well as psychiatric syndromes that occur in patients with SLE without an alternative cause. Seizures are among the most common and distinct manifestations of NPSLE [13]. PRES originally described in 1996 by Hinchey et al. [14] is an uncommon and non-specific presentation likely caused by vasogenic subcortical brain edema, commonly in the posterior parieto-occipital lobes [15]. It is a syndrome consisting of headache, altered mental status, and seizures with or without loss of vision. Peripheral Neuropathy in SLE is uncommon, and very few studies have noted such a presentation. This can result in a delay in diagnosis and treatment. In the lower extremities, the most commonly involved nerves have been found to be the sural nerve (55.2%) and the peroneal nerve (53.9%). And the most common presentation noted was distal axonal sensory or sensory-motor polyneuropathy [16]. In our patient, neurological involvement in the form of PRES helped us to establish the diagnosis of NPSLE.

Previous studies have demonstrated that patients with SLE retinopathy tend to have significantly lower hemoglobin levels and lower serum C3 and C4 concentrations [2]. A study by Seth et al. has shown that immunological markers, including antibodies against U1 ribonucleoprotein (U1RNP), SS-A, Ro-52, Ribosomal P, and Anti-Sm antibody, were higher among those SLE patients with retinopathy [17]. ANA testing may initially be seronegative and later turn positive on serial immunofluorescence testing using HEp-2 cells, owing to factors such as antigen-deficient substrates, antigen leaching, concurrent immunosuppressive therapy, and persistent renal protein loss [18]. The laboratory findings in our case were consistent with those reported in these studies.

SLE and ocular TB may share systemic and retinal features such as vasculitis, hemorrhages, and vascular occlusions; however, SLE tends to involve both the arteriolar and venular systems [19], whereas TB predominantly affects the venous circulation, although arteriolar involvement is noted less commonly [20]. An atypical confounding presenting feature that led to a diagnostic dilemma in our case was peripheral choroiditis on fundus examination. Although peri- or sub-vascular choroiditis is a common feature of TB vasculitis [20], we hypothesize that in our case, inflammation following active SLE vasculitis might have damaged the retinal pigment epithelium and choroid, as evident by vessel pruning proximal to the lesions and persistent hypocyanescence on ICGA, reflecting underlying choroidal hypoperfusion. In our case, the patient initially presented with BRAO and retinal vasculitis, which was misinterpreted as a manifestation of ocular TB, a common diagnostic dilemma in TB endemic regions like India. The initial diagnosis of ocular TB was based on systemic symptoms such as evening pyrexia, weight loss, and pulmonary findings, which clinically overlapped with constitutional features of SLE. However, the lack of microbiological confirmation, non-specific HRCT findings, and negative BAL cultures eventually led to a reconsideration of diagnosis and the withdrawal of ATT. This further prompted us to re-evaluate and reinvestigate our patient in search of an alternative diagnosis.

The revision of diagnosis to SLE in our case was based on a thorough systemic and multidisciplinary investigation fulfilling the diagnostic criteria proposed by the European League Against Rheumatism/American College of Rheumatology (EULAR)/ACR) 2019 classification criteria for SLE [21]. With positive ANA as an entry criterion and a score of 25 determined by additive criteria - 2 for fever, 4 for thrombocytopenia, 5 for seizure, 4 for subacute cutaneous lupus, 4 for low C3 and C4 levels, and 6 for SLE-specific antibodies such as anti-double stranded (Anti-ds)DNA antibody and Anti-Smith antibody, our case was a confirmed case of SLE.

## Conclusions

This challenging case underscores the diagnostic complexity of SLE in tuberculosis-endemic regions, where overlapping systemic and ocular features may mimic tuberculosis. The presentation of BRAO in a young patient, though rare in SLE, should prompt consideration of an underlying autoimmune pathology, especially when supported by systemic features and immunological markers. Our case also highlights the possibility of lupus retinopathy as an early ophthalmic manifestation of SLE, which may herald underlying severe systemic involvement. This further reinforces the need for timely and comprehensive immunological evaluation to ensure accurate diagnosis and prompt appropriate management.

## References

[1] Silpa-archa S, Lee JJ, Foster CS (2016). Ocular manifestations in systemic lupus erythematosus. Br J Ophthalmol.

[2] Bashiri H, Karimi N, Mostafaei S, Baghdadi A, Nejadhosseinian M, Faezi ST (2021). Retinopathy in newly-diagnosed systemic lupus erythematosus: should we screen for ocular involvement?. BMC Rheumatol.

[3] Meng L, Wang Y, Yang Z (2024). Ocular fundus changes and association with systemic conditions in systemic lupus erythematosus. Front Immunol.

[4] Read RW, Chong LP, Rao NA (2000). Occlusive retinal vasculitis associated with systemic lupus erythematosus. Arch Ophthalmol.

[5] Klinkhoff AV, Beattie CW, Chalmers A (1986). Retinopathy in systemic lupus erythematosus: relationship to disease activity. Arthritis Rheum.

[6] Graham EM, Spalton DJ, Barnard RO, Garner A, Russell RW (1985). Cerebral and retinal vascular changes in systemic lupus erythematosus. Ophthalmology.

[7] Luboń W, Luboń M, Kotyla P, Mrukwa-Kominek E (2022). Understanding ocular findings and manifestations of systemic lupus erythematosus: update review of the literature. Int J Mol Sci.

[8] Cutrufello NJ, Karakousis PC, Fishler J, Albini TA (2010). Intraocular tuberculosis. Ocul Immunol Inflamm.

[9] Weckerle CE, Niewold TB (2011). The unexplained female predominance of systemic lupus erythematosus: clues from genetic and cytokine studies. Clin Rev Allergy Immunol.

[10] Mahjoub A, Dlensi A, Ben Abdesslem N (2022). Lupus choroidopathy: a case report. J Fr Ophtalmol.

[11] Zhang L, Guan C, Ye Z, Lu Y (2022). Unilateral branch retinal artery occlusion in a patient with systemic lupus erythematosus: a case report. Medicine (Baltimore).

[12] Taraborelli M, Cavazzana I, Martinazzi N (2017). Organ damage accrual and distribution in systemic lupus erythematosus patients followed-up for more than 10 years. Lupus.

[13] Andrade RM, Alarcón GS, González LA (2008). Seizures in patients with systemic lupus erythematosus: data from LUMINA, a multiethnic cohort (LUMINA LIV). Ann Rheum Dis.

[14] Hinchey J, Chaves C, Appignani B (1996). A reversible posterior leukoencephalopathy syndrome. N Engl J Med.

[15] Hinduja A (2020). Posterior reversible encephalopathy syndrome: clinical features and outcome. Front Neurol.

[16] Florica B, Aghdassi E, Su J, Gladman DD, Urowitz MB, Fortin PR (2011). Peripheral neuropathy in patients with systemic lupus erythematosus. Semin Arthritis Rheum.

[17] Seth G, Chengappa KG, Misra DP (2018). Lupus retinopathy: a marker of active systemic lupus erythematosus. Rheumatol Int.

[18] Tiwary AK, Kumar P (2018). Paradigm shift in antinuclear antibody negative lupus: Current evidence. Indian J Dermatol Venereol Leprol.

[19] Babu K, Nanda S, Hegde P, Rao AP, Jois R (2023). Posterior segment involvement in systemic lupus erythematosus - a series from South India. Indian J Ophthalmol.

[20] Basu S, Talluri RA, Kelgaonkar A (2025). Tubercular retinitis and retinal vasculitis: manifestations of a shared disease spectrum. Ocul Immunol Inflamm.

[21] Aringer M, Costenbader K, Daikh D (2019). 2019 European League Against Rheumatism/American College of Rheumatology classification criteria for systemic lupus erythematosus. Ann Rheum Dis.

